# LionVu: A Data-Driven Geographical Web-GIS Tool for Community Health and Decision-Making in a Catchment Area

**DOI:** 10.3390/geographies3020015

**Published:** 2023-04-18

**Authors:** Nathaniel R. Geyer, Eugene J. Lengerich

**Affiliations:** 1 Department of Public Health Sciences, Penn State College of Medicine, Penn State University, Hershey, PA 17033, USA; 2 Penn State Cancer Institute, Hershey, PA 17033, USA

**Keywords:** QGIS, Leaflet, GIS, big data, web-based GIS, cancer, catchment area

## Abstract

In 2018, the Penn State Cancer Institute developed LionVu, a web mapping tool to educate and inform community health professionals about the cancer burden in Pennsylvania and its catchment area of 28 counties in central Pennsylvania. LionVu, redesigned in 2023, uses several open-source JavaScript libraries (i.e., Leaflet, jQuery, Chroma, Geostats, DataTables, and ApexChart) to allow public health researchers the ability to map, download, and chart 21 publicly available datasets for clinical, educational, and epidemiological audiences. County and census tract data used in choropleth maps were all downloaded from the sources website and linked to Pennsylvania and catchment area county and census tract geographies, using a QGIS plugin and Leaflet JavaScript. Two LionVu demonstrations are presented, and 10 other public health related web-GIS applications are reviewed. LionVu fills a role in the public health community by allowing clinical, educational, and epidemiological audiences the ability to visualize and utilize health data at various levels of aggregation and geographical scales (i.e., county, or census tracts). Also, LionVu is a novel application that can translate and can be used, for mapping and graphing purposes. A dialog to demonstrate the potential value of web-based GIS to a wider audience, in the public health research community, is needed.

## 1. Introduction

Over fifty years ago, the National Cancer Act established a program for cancer research and demonstration centers to be administered by the National Cancer Institute (NCI) [1]. In 2012, the NCI Funding Opportunity Announcement (FOA), as part of the P30 Cancer Center Support Grant, required that designated cancer centers establish and describe their catchment areas and document research that addresses cancer burden, risk factors, incidence, morbidity, mortality, and inequalities [2]. In 2016, the focus was expanded to include community outreach and engagement [3]. However, the FOA provided limited guidance and was difficult for reviewers to review the grant applications [4]. Moreover, the catchment area can be defined by the cancer center, which can range from smaller than a single county to over 200 counties, with the total NCI catchment area encompassing approximately 2.4 million square miles (6,215,971,464,810 m^2^) [1].

NCI’s Catchment Area Initiative increased the need for using Geographic Information Systems (GIS). Research using GIS is critical for cancer prevention and control. Between 1971–2016, the peer-reviewed literature has at least one affiliation in one or more author within a NCI-designated Cancer Centers [5]. Cancer data visualization tools needs to include academic, governmental, or community stakeholders with some knowledge of GIS, while safeguarding ethical concerns [6]. Specifically, the Health Insurance Portability and Accountability Act (HIPAA) limits the use of personally identifiable health information (PHI) for research purposes. Common examples of PHI include names, dates (except year), telephone numbers, Web addresses, and geographic data [7]. Therefore, geographical data below a state-level, may be protected health information and safeguarded by HIPAA [8]. Until recently, educators at public health graduate schools have not taught GIS, limiting the emergence of combined Internet-based geospatial technologies and health-based applications. However, with the NCI Catchment Area Initiative, the gap between software-based and web-based GIS tools, in public health, is narrowing.

Pennsylvania has 67 counties with almost 45% rural (30 counties). In addition, Pennsylvania includes 52 Appalachian counties, 28 counties in the Penn State Cancer Institute catchment area, and 19 classified as both catchment area and Appalachian counties out of 67 total counties. In Pennsylvania, there are no rural (i.e., RUCC > 3) non-Appalachian counties in the state; therefore all 30 rural counties and 22 urban counties fall in the Appalachia Subregion [9], with one-fourth (107 out of 423 counties) of are rural within 13 states. In general, Appalachia’s counties lagged in areas in most indicators, with populations in vulnerable economic positions pre-COVID-19 [10,11]. Since approximately 78% (52 Counties) of Pennsylvania are within Appalachia shows the need for a web-GIS tool.

**Figure 1.**
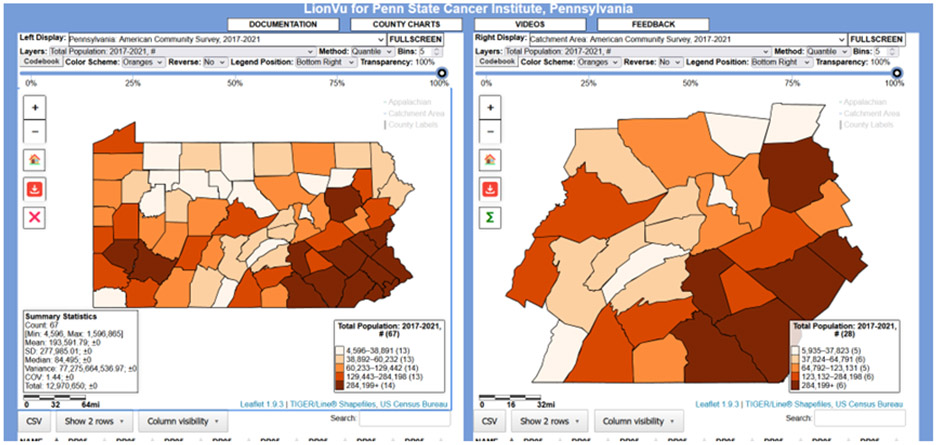
Screenshot of LionVu with the Summary Statistics turned on in the left display. Accessed on 20 March 2023 (1 mi ≅ 1610 m).

Originally, LionVu, consists of a web-based GIS tool that displays spatial data in Pennsylvania and the 28-county catchment area of the Penn State Cancer Institute, was developed using ArcGIS Online. Since ArcGIS Online is proprietary, we found that moving to a HIPAA-protected server a payment is necessary, and it was challenging to add newer data to the tool. In response, LionVu was redesigned using Leaflet JavaScript, and a usability assessment was completed [12]. Leaflet uses the Web Mercator projection, which is based on WGS84 and remains the de facto projection for web mapping applications [13]. Figure 1 shows the most recent version of LionVu, which is publicly available online at (https://app-phs.hmc.psu.edu/lionvu/), accessed on 20 March 2023.

LionVu datasets are a single point in time, so it is critical for only mapping the latest information using an automated process. The challenge of updating the data was the extensive preprocessing required, such as merging to a geographical file and converting to Leaflet JavaScript file format. Previously, our team merged a CSV, or text file with a geographical file manually into a GeoJSON format. is an Open Geospatial Consortium (OGS) standard that holds geographical data in a JSON format [14]. Leaflet JavaScript best utilizes GeoJSON as a JavaScript variable; meaning that we had to manually add a JavaScript variable name with a semicolon at the end, which was tedious for multiple datasets. Moreover, we found that manual conversion from GeoJSON file to Leaflet JavaScript using Notepad+ caused extensive manual review and was prone to human error. We found that data was easily corrupted, by manual review, rendering files useless, which was especially problematic when working with 21 datasets and two geographies. Comparatively, QGIS has the flexibility to handle multiple python libraries and it can be reused by others in the GIS field [15]. The final product is a JavaScript file, which can be uploaded, to a hosted HIPAA-protected cloud-based Server maintaining LionVu, using FileZilla.

The three aims of this paper are: (a) to describe the process-flow of updating LionVu, including the methodology of the Data to Leaflet plugin graphical user interface with all its functionality; (b) to provide two demonstrations of using LionVu to map and chart publicly available datasets collected from various sources, including the United States Census Bureau and Pennsylvania Department of Health; and (c) to compare the functionality of LionVu to ten other public-health related web GIS tools. Demonstrations will include both area (i.e., population density per dentist and oral cancer) and point-level (i.e., finding hospitals, federally qualified health centers, and medically underserved areas in Dauphin County, Pennsylvania) using spatial vector data integrated into LionVu. The main functionalities of LionVu are all publicly available, using open-source JavaScript libraries.

## 2. Data to Leaflet JavaScript Plugin

**Figure 2.**
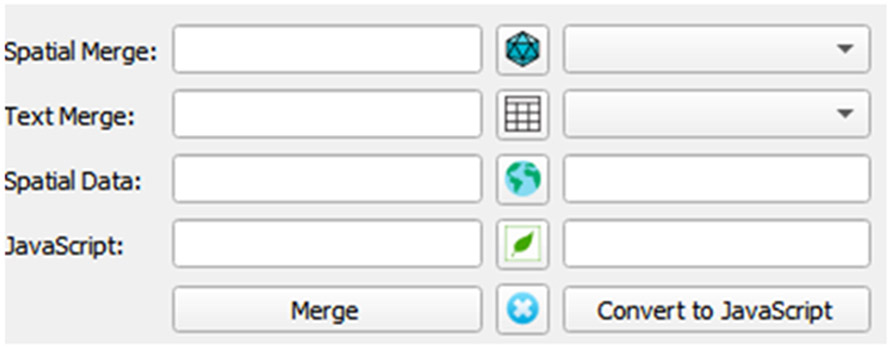
CSV to Leaflet Plugin graphical user interface.

In 2021, we developed the Data to Leaflet JavaScript QGIS plugin, specifically for converting CSV or text files to GeoJSON format, and then a JavaScript variable to work with Leaflet JavaScript. Both Leaflet and QGIS are GIS software that have a large community of users and developers. To automate the process of using CSV or text file and/or spatial data sets (e.g., KML, SHP, GeoJSON, or GeoPackage) inputs to a Leaflet JavaScript output, we created a graphical user interface accessible through the plug-in dropdown menu. In the plug-in the first two rows are for merging the two files, and the third and fourth rows create the Leaflet JavaScript file in either point, line, or polygon geometries (Figure 2).

**Figure 3.**
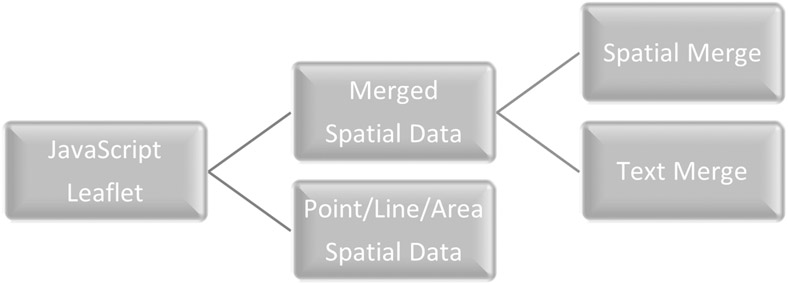
Flow chart of CSV to Leaflet QGIS Plugin.

To use the QGIS plug-in, we uncovered the necessity for the spatial data and spatial merge data, which was projected using the WGS84 instead of the NAD83, commonly used in North America and saved as a GeoJSON format. After loading the spatial data, a list or file name appears on the right side. The text merge option requires that the data be either in CSV or text format to create a list of field names that can be set as the variable used in the CSV merge tool. The merge button is used to complete the join, and only links the data where the same values and type are similar. Once the data has successfully merged and appeared in the QGIS layer listing and appears in the third row, clicking on Convert to JavaScript converts the layer to a Leaflet JavaScript format. Clicking on the globe in the middle of the third row instead of merge button will directly create the JavaScript file from the spatial dataset without merging, and then clicking on the Leaflet button converts the JavaScript file to JSON format, which allows JavaScript libraries (i.e., Leaflet, DataTables, Chroma, and Geostats) to utilize the data (Figure 3).

Conceptually, our team created the QGIS plugin using Python 3, with five dependencies (i.e., OS, CSV, JSON, RE, PyQt5) and QGIS functionality. More specifically, we used CSV and OS libraries to import the CSV or text file into QGIS and create a list of variable names; and OS and JSON libraries to export the GeoJSON into a minified Leaflet JavaScript format. The other two libraries added minor parts to the plugin to make the tool smarter and more able to work for vector data of points, lines, or polygon geometries. A minified JavaScript file removes unnecessary characters without changing its functionality, allowing LionVu to run efficiently. Our team evaluated the plugin using QGIS 3.28.4 on Windows 11, using either county or census tract geographies (Supplementary Material S1). The QGIS reference manual should be consulted to properly install the plugin to QGIS 3+ [16].

Our team used the Data to Leaflet Plugin, to standardize the geographic files for speeding up the merging with a CSV or text file. For example, the 2023 LionVu release included both the county and census tracts data sets from the 2022 United States Census Bureau’s TIGER/Line SHP program. Using QGIS 3+, we reprojected from NAD83 to WGS84 and filtered out an approximately 735 mi^2^ (1,903,641,260 m^2^) of a single census tract (i.e., GEOID: 4204990000) in Erie County that was completely in Lake Erie. To make the spatial data smaller, we stripped all variables except county name. Since LionVu includes both Pennsylvania and the Penn State Cancer Institute catchment area geographies, we created two GeoJSON files for both the county and census tract geographies.

## 3. LionVu Functionality

The 2023 redesign of LionVu, utilized the Leaflet, jQuery, Chroma, Geostats, DataTables, and ApexChart JavaScript libraries, and a feedback form, on 15 February 2023 (Table 1).The end-user can: display the data on the website via the Leaflet JavaScript mapping library; pick a Color Brewer 2.0 schema using Chroma JavaScript library choose the number of bins (i.e., 3–20) [17,18]; a classification method (i.e., quantiles, natural breaks, equal intervals, standard deviation, arithmetic progression, geometric progression, maximum breaks, and unique values); and utilize the Geostats JavaScript Library, except for the manual classification method, which cannot be applied to a dropdown menu. Additionally, on the web-GIS tool, the end-user has either four or five buttons to adjust the zoom (i.e., in, out, home), to download the map to image, and to display the secondary legend (in choropleth maps only). On the top-right, there are buttons to turn on county labels and boundaries (e.g., catchment area, Appalachia, and county [in census tract choropleth maps only]) to add more detail to the web map. The end-users can see the table of the datasets below the maps via jQuery and its DataTable plugin. The end-user can click on the County Charts button to create two bar charts and one scatter plot of the chosen values for county-level datasets using ApexCharts Library. Finally, the end-user can also use the feedback form to send emails to the developer and other end-users using PHP syntax.

By default, LionVu maps American Community Survey’s population estimates for Pennsylvania in the left-side display and the Penn State Cancer Institute catchment area in the right-side display. The other defaults include Oranges Color Brewer 2.0 schema, 5—bin values, quantiles classification, no reverse color schema (i.e., light to dark), primary legend position on the bottom right, 100% transparency, no summary statistics, and a data table below only showing two rows per data. The codebook button downloads a CSV file for each of the relevant datasets selected in the two dropdown menus. As shown in Figure 1, the display also includes a secondary legend, which is by default turned off, that contains simple summary statistics (e.g., count, minimum, maximum, mean, standard deviation [SD], median, variance, coefficient of variation [COV], and total) for the mapped layer.

In the Healthcare Facilities and Underserved Area section, we included the HRSA-defined underserved area component boundaries (i.e., dental, medical, mental, and primary care) and individual-level data from various health care providers (i.e., Penn State Health, ambulatory surgery centers, federally qualified health centers, hospitals, mammography, and rural health centers). Since this section includes 10 data sets and no choropleth maps, we added only 10 codebook buttons appear with captions at the bottom of the page. We found that the rest of the providers contain both Pennsylvania and other catchment area geographies. However, since the Penn State Cancer Institute catchment area is specific to our group and consists of 28 counties, we were required to use street and zip code geocoding using SAS 9.4 [19]. The primary reason is the special case where Bethlehem, Pennsylvania is in both Lehigh and Northampton Counties but only Lehigh County is in the Penn State Cancer Institute catchment area.

Currently, the charting functionality in LionVu is for county-level datasets only and utilizes two bar charts by county for a selected field, then creates a scatter plot of the given field on the left display as the *x*-axis and right display as the *y*-axis field. The challenge was keeping the load time down and preventing out-of-memory errors, which slows down the charting functionality in LionVu. In the case where a non-county dataset exists on either the left or right display, LionVu charts the default American Community Survey’s population estimates, with a sweet alert to notify the user about the issue.

Other LionVu functionality includes a 5-min video, documentation, and a feedback form, allowing an end-user to send an email providing feedback on improving the website, while filtering out spam. Our team developed a HTML documentation file on the website supplying additional information on methods useful for guidance, including attributions to external sources, and hyperlinks to download the QGIS plugin and geography files. We made sure to allow the end-user the ability to download the documentation as one file format: PNG image, CSV, PDF, or GeoJSON. PNG images include most maps and charts. CSV files include the data sets and the codebooks. PDF files include the documentation and Pennsylvania EJSCREEN, which was too big for the Leaflet plugin to save as a PNG image file. We also included a GeoJSON with Pennsylvania and the Penn State Cancer Institute catchment area geographies to aid with future updates (Supplementary Material S2).

## 4. Data Sources

LionVu includes 21 data sources in the 2023 LionVu release and allows for various kinds of visualizations and analyses of these data sources. We categorized the downloaded data sets into four main groups:

1. **Health outcomes and access to care data**: measures concerning the health of the community or access to care, such as cancer incidence, early stage, late state, survival, mortality, County Health Rankings, County Health Profiles, and health providers.
2. **Socioeconomic and environmental exposures data**: economic indicators, policy, population behavioral changes, environmental health justice, social vulnerability, and health insurance.
3. **Multiple**: County Health Ranking, which consists of health outcome, access to care, and both socioeconomic and environmental exposure information.
4. **Geographical data**: county and census tract geographies, county centroids, county boundaries, catchment area, and Appalachian County designation.

Table 2 outlines the main data sources used in LionVu as of 20 March 2022, including the organization, topics, year, and geography. The organizations included in LionVu, are the Pennsylvania Department of Health, United States Census Bureau, County Health Profiles, and other data sources (i.e., FDA. HRSA, CDC, EPA, and ARC). We saved the data sets (i.e., geographies, catchment data, Pennsylvania data, and point or individual-level data) and codebooks used in LionVu for Pennsylvania and the Penn State Cancer Institute catchment area geographies, as either CSV or GeoJSON formatted files and accessed on 27 February 2023 (See Supplementary Material S2).

## 5. Results

This section focuses on two demonstrations and then compares LionVu to other public health-related web-GIS tools. The two demonstrations are based on choropleth map and charting functionality of LionVu and used the Healthcare Facilities and Underserved Area section to find providers and underserved areas in one county. Demonstration one used area data dealing with population density per dentist and oral cancer incidence, which uses both the mapping and charting functionality in LionVu. The second demonstration used individual-level data in LionVu to find hospitals, federally qualified health centers, and medical underserved areas within Dauphin County, Pennsylvania, which is one of the Penn State Cancer Institute catchment area counties. We included a comparison between LionVu and ten other public health related web-GIS tools.

### 5.1. Area Data Example: Population Density per Dentist vs. Oral Cancer Incidence

**Figure 4.**
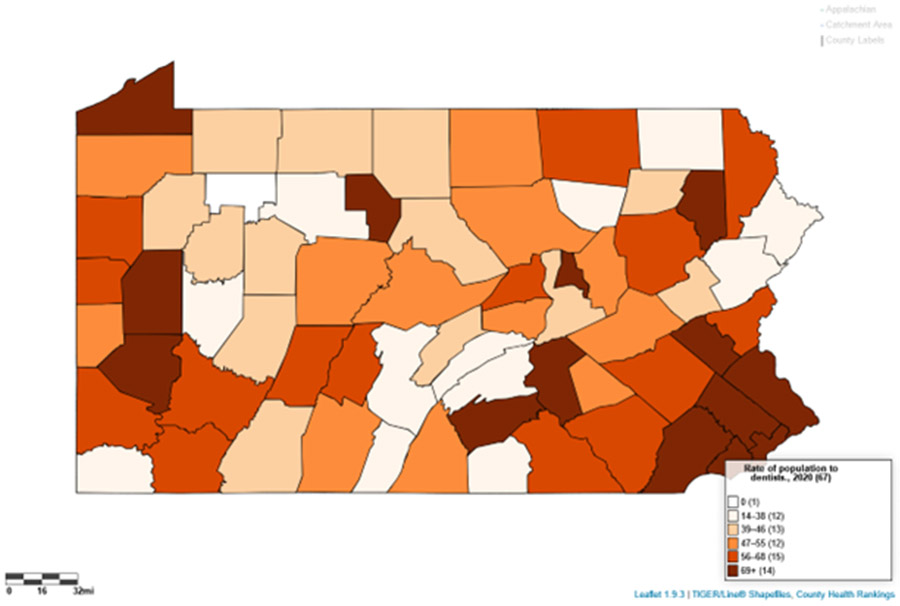
Population density per dentist in Pennsylvania, 2020 (1 mi ≅ 1610 m).

**Figure 5.**
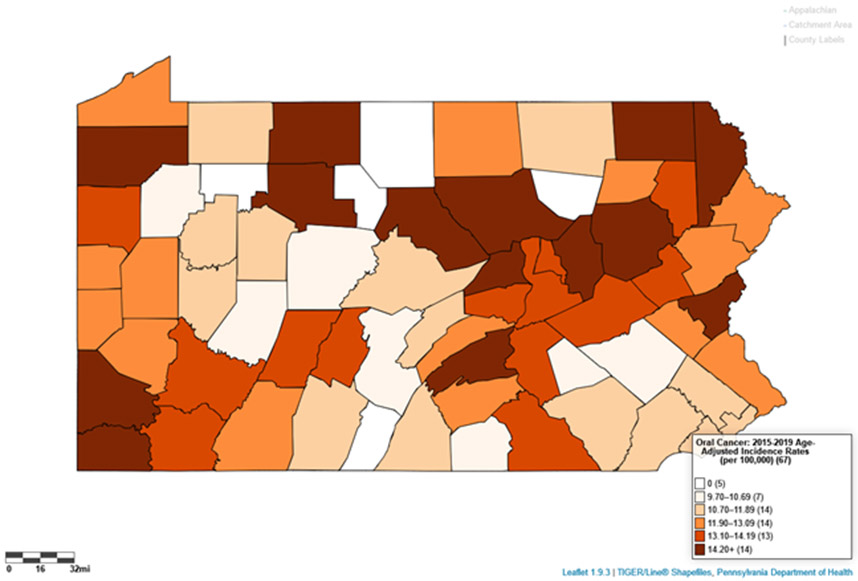
Oral cancer incidence rates, 2015–2019 (1 mi ≅ 1610 m).

**Figure 6.**
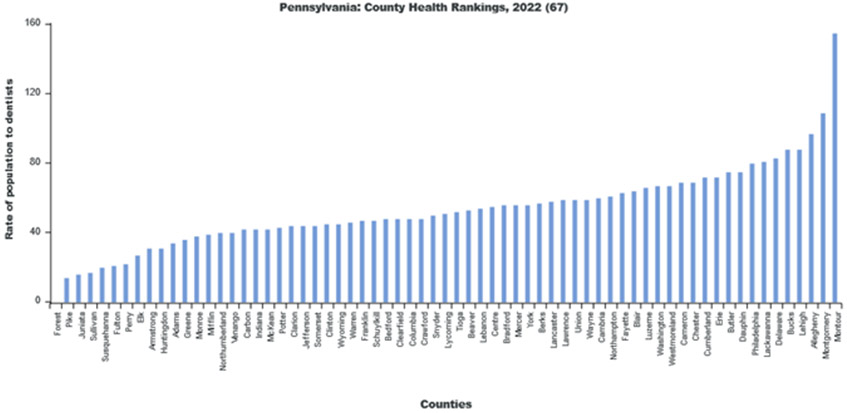
Bar chart of the population density per dentist 2020 per Pennsylvania County.

**Figure 7.**
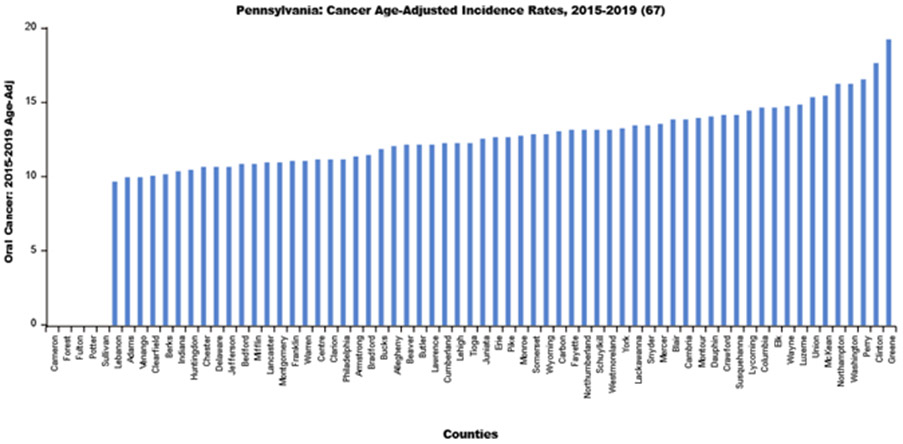
Bar chart of the 2015–2019 oral cancer incidence rate per Pennsylvania County.

**Figure 8.**
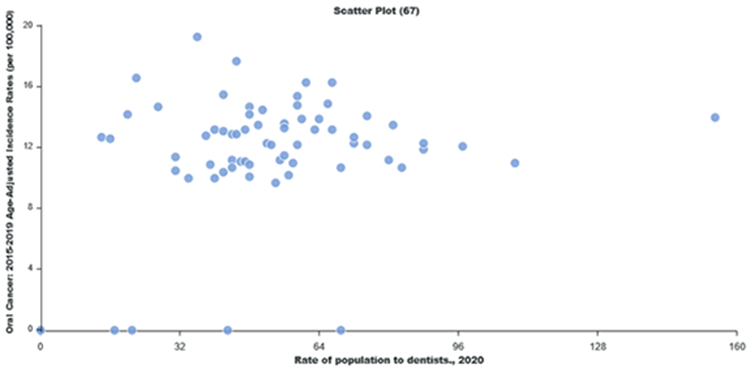
Scatter plot of prevalence of oral cancer incidence rate in comparison with population density of dentists.

Using LionVu’s side-by side functionality, it is possible to put the population density per dentist (Pennsylvania: County Health Ranking) on the left side, oral cancer incidence (Pennsylvania: Cancer Incidence Age-Adjusted Rates), on the right side, and then download both images to PNG format (Figures 4 and 5). The default 5—bin, quantiles, and orange color schema choropleth map of county showed a potential comparison between the two figures. The county charts functionality enables the display of the county percentages and rates in two bar charts (Figures 6 and 7) and one scatter plot (Figure 8). This demonstration shows that counties that have a lower population density per dentist also have an increase in oral cancer. The county charts show that Montour County is an outlier because of both a high population density per dentist and oral cancer. Cameron, Forest, Fulton Potter, and Sullivan Counties had a zero oral cancer incidence because the Pennsylvania Department of Health did not display the rates due to small cell counties, which suppressed the results, due to HIPAA issues. Forest County also had zero population density in the County Health Ranking data due to similar HIPAA issues. We believe that the actual rates are closer to the rates of the neighboring counties.

### 5.2. Individual-Level Data Example: Finding Hospitals, Federally Qualified Health Centers, and Medical Underserved Areas in Dauphin County, Pennsylvania

**Figure 9.**
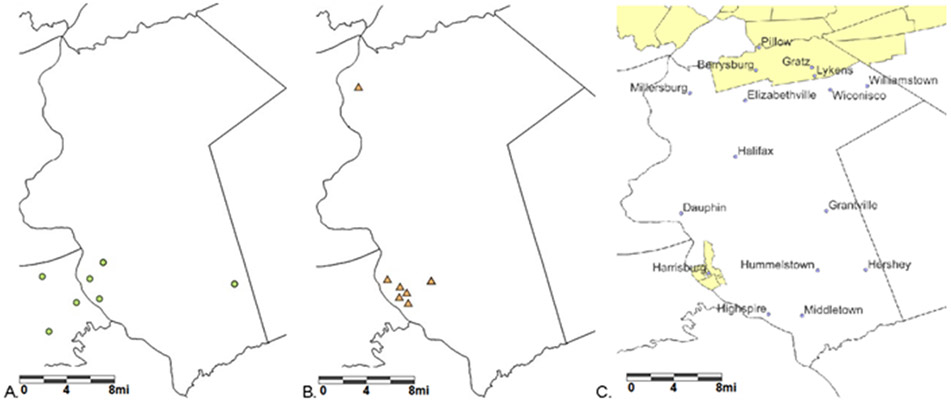
Providers in Dauphin County: (**A**) hospitals; (**B**) federally qualified health centers; and (**C**) medical underserved areas with cities and boroughs (1 mi ≅ 1610 m).

Dauphin County in southcentral Pennsylvania, an urban non-Appalachian county, has severe inequalities. The Southwest of Dauphin County contains the state capital Harrisburg, which is separated from western Pennsylvania counties by the Susquehanna River, Harrisburg has a population of 50,135 people (34% white, 50% black, 25% Hispanic), of which 9.9% have no health insurance, and 28% live in poverty [20]. The southeastern area near Hershey (a census-designated place) has 13,858 people with higher wealth and more health care providers than other parts of the county, which includes 78% white, 2% black, 12% Hispanics, 2% have no health insurance, and 9% live in poverty [21]. The northeastern part of the county, near Lykens, has 1932 people, of which 80% are white, 8% have no health insurance, and 30% are in poverty [22]. Figure 9 shows that there are four hospitals in Dauphin County (three in the Harrisburg area and one in Hershey), seven federally qualified health centers (six in the Harrisburg area and one in Millersburg), but only two medical underserved areas—one in the northeast (i.e., Berrysburg, Gratz, Lykens, and Pillow) and one in the Harrisburg area. The data for the cities and boroughs come from de-duplicating the SAS 9.4 zip code dataset and using QGIS 3.28.4. These three maps suggest that in Dauphin County, Pennsylvania, there is a need for more medical services in the Harrisburg area and in northeastern parts of the county, including Berrysburg, Gratz, Lykens, and Pillow.

### 5.3. LionVu Comparison to Other Public Health-Related Webmaps

In 2014, Luan and Law did a comprehensive comparison of 27 public health-related web maps [23]. In 2023, the authors of the current paper found that out of the 27 web maps referenced, only 8 were still regularly updated. For example, the Pennsylvania West Nile program is still live but not being regularly updated. Another web map, the Periscope Atlas, was newly created for the COVID-19 epidemic in Europe [24]. Both authors [23,24] did not cite Pennsylvania Department of Health’s Enterprise Data Dissemination Informatics Exchange, but we reviewed it for this assessment. The Pennsylvania Department of Health’s Enterprise Data Dissemination Informatics Exchange was not cited in either study but was reviewed for this assessment. Table 3 shows the comparison of ten different GIS public health websites. CDC’s cancer statistics website contains a national and state web map of cancer-related data. CDC’s Diabetes Interactive Atlas uses bubble and candlestick charts specific to diabetes. CDC Heart and Stroke Prevention has a comprehensive dataset on the conditions, health determinants, and environmental factors. CDC’s oral health map is updated every two years. The Pennsylvania Department of Health’s Enterprise Data Dissemination Informatics Exchange had various health related data, from which the data easier to download, but it is more difficult to fit the data and to create a bivariate display for more than one variable at multiple time periods. EpiScanGIS is a German web map for meningococcal disease, containing both point and area data of seropositive populations, and is updated weekly, unlike LionVu, which is mostly area data and is updated annually. HealthMap is global, focused on influenza and COVID-19, and uses ArcGIS Online, time series data, and symbols. The Kentucky Cancer Registry was easier to drill down than LionVu for more specific cancer diagnoses, including rarer types of cancer, but it only has data for one state with no other data sources. Florida Health Charts has state, census tract, zip code, and county geographies, with data and functionality comparable with the Pennsylvania Department of Health. Finally, the Periscope Atlas was developed for COVID-19 and has more multivariate analysis than LionVu. All ten web-GIS tools examined had at least one choropleth map.

## 6. Discussion

The three aims included: (a) describing the process-flow of updating LionVu, including the methodology of the CSV to Leaflet plugin graphical user interface with all its functionality; (b) providing two demonstrations using LionVu to map and chart publicly available data collected from County Health Ranking and Pennsylvania Department of Health; and (c) compare LionVu with ten other public health related Web-GIS tools. For the first aim, the Data to JavaScript plugin automated data processing and minimized manual review. The second aim included maps, bar charts, and a scatter plot to compare the population density per dentist with oral cancer incidence, which shows an increasing trend in the scatter plot. Furthermore, the health care providers in Dauphin County, Pennsylvania showed how LionVu data can fit a hypothesis-driven assessment. The third aim suggests that LionVu has functionality that is comparable to the ten other web-GIS tools examined. The discussion section overviews this LionVu assessment, the lessons learned, the strengths and limitations, and further recommendations.

### 6.1. LionVu Assessment

LionVu, as presented in this paper, was the result of using secondary data and various JavaScript libraries, functions, and results. Thanks to the 21 datasets included, LionVu makes it easier for community health members in Pennsylvania to implement this information into research projects, teaching, and other uses. This project provides a low-cost strategy to implement a web-GIS tool, with the possibility of adding newer functionality to improve mapping in the future. Other teams could replicate LionVu’s tools, depending on knowledge about markup, scripting languages, JavaScript libraries [25], and spatial analyses techniques [26]. Moreover, a basic knowledge of the spatial data standards and open-source software, such as QGIS, Leaflet, or FileZilla, is necessary to relieve the programmer of developmental works due to ready-made functions and classes [25]. Unlike proprietary GIS software, QGIS and other open-source tools used in LionVu reads most data formats, and has one data license, no vendor lock-in, is free to use and is customizable, has improved reliability and quality, requires less preparation time, and has better rendering capacities [15]. For example, QGIS has a built-in function that quickly creates directory tiles processed by Leaflet, using a landscape model that converts spatial data into GeoJSON format that can be read by Leaflet as a JavaScript variable [27]. These features make open-source solutions the way of the future.

The demonstration of the comparison of population density per dentist and oral cancer shows that there may be a significant multivariable association between the two with a spatial autocorrelation, which is outside the scope of this study. The maps and charts show that Montour, Sullivan, and Fulton counties, which are in the Penn State Cancer Institute catchment area, are outliers in this analysis. There appears to be an inverse relationship between dental care and oral cancer incidence. In Figure 8, we found, after removing the outliers, that an increase in the population density per dentist correlate with the increase in oral cancer incidence in Pennsylvania. Since the source data could be downloaded within LionVu via a CSV output, it is possible to use spatial analysis approaches. For example, we completed a previous auto-correlation analysis looking at ambulatory surgery centers in Pennsylvania in relation to colorectal cancer mortality, using data from LionVu [28].

The example of using point-level maps to determine providers in Dauphin County, Pennsylvania, shows the potential for LionVu to support hypothesis-driven research. The biggest challenge is HIPAA preserving confidentiality and doing research. In LionVu, we added pop-ups for each health care provider, and could also download the datasets and zoom in the data. However, to create a reference map for demonstration purposes (see Figure 9), we had to add the cities and boroughs in QGIS. LionVu is a hypothesis-driven tool to supplement and not conduct research. If an end-user wants to do advanced analysis or, for example add cities and boroughs to a map of Pennsylvania, one can use supplemental software such as ArcGIS, QGIS, R, GeoDa, SAS, and others.

Based on the ten different web maps examined (see Table 3), the majority only have data on one type of condition. This is often the result of teams developing products in an internal silo, which limits the content, or only performing basic analyses for a restricted set of variables [29]. For example, if a person needs national data from CDC for Cancer, Diabetes, Heart Disease and Stroke, or Oral Health a person must go to four web maps for national data, since they do not zoom within a state, nor do they allow cross-disease comparisons. In LionVu, the system is limited to the availability of publicly accessible data and server space. A person could theoretically download cancer (e.g., mortality, incidence, and survival) from the three dropdowns; diabetes, heart disease, and stroke data from the county health profile section; and oral health from the County Health Rankings subset, without leaving the website.

### 6.2. Lessons Learned

One of the first lessons learned is how to justify web mapping with big data. Big data refers to tools that extract information based on four factors: large volume (size), high velocity (time frequency), large variety (data types), and veracity (quality). Some of the potential benefits of big data to public health are determining risk factors that lead to diseases, such as cancer, and that generate new knowledge, improve clinical care, and streamline health surveillance [30]. When using GIS to present public health data, there are issue that restrict data availability, such as HIPAA [7], but one also has to watch out for data heterogeneity, data security, transparency, and fair use of data [30]. In the case of LionVu, we are providing hypothesis-driven research that uses smarter GIS functionality in ways that can utilize big data sources (see Table 2).

A second lesson is justifying LionVu in terms of systems development. To develop a successful web-GIS public health tool, it is important to focus on health data collection, user interface design, maps and charts, system maintenance, privacy issues, cost, and training [23]. LionVu has over 21 data sources, updates data annually, has a side-by-side user interface, contains both maps and charts, is maintained on a HIPAA protected server, uses open-source tools, and contains a short video for training purposes. However, in terms of training and reaching out to the target audience, the clinical, educational, and epidemiology researchers, it is still a working progress.

The third lesson is to develop a system that reaches a public health audience. Currently, public health and clinical graduate schools do not teach geospatial methodology, such as mapping software and web-based GIS tools, even though there is a need for ecological research. Because of legislations such as HIPAA, many individual-level researchers and staff are struggling to make sense of geographical data [31], leading to misinterpretation [32]. For example, the CDC printed choropleth maps that were not data normalized, where counts were not standardized based on population size using a rate, density, or percentage, and that underscored the impact of COVID-19 in areas with fewer cases and small populations, leading to poor policy decisions [33]. One strategy to correct these issues is teaching geospatial thinking and reasoning skills to undergraduate students in higher education. Geospatial thinking and reasoning skills include developing a geospatial research question, creating a geospatial data visualization, performing a geospatial data analysis, supporting a geospatial explanation, and justifying the argument and claims [34]. In this way, by the time the student progresses into graduate or medical schools, they are more likely to be familiarized with GIS and make better sense of spatial data.

### 6.3. Strengths and Limitations

Data to JavaScript is the first QGIS plugin to integrate QGIS and Leaflet in a way that automates the processing while minimizing manual review. It currently works best on vector data with a predetermined projection because reprojecting is not currently an option but is an area of development in the future. Another related issue is that although the format of the spatial data is flexible, the text data must be either CSV or text files, which excludes other formats, due to a QGIS import issue. Despite these limitations, the QGIS plugin could be a valuable resource to people who work with both QGIS and Leaflet.

The strength of LionVu is the ability to view images side-by-side, as two univariate maps, and to produce bivariate plots or charts, such as in our example where we have both the Penn State Cancer Institute catchment area and Pennsylvania geographies. LionVu is a quick study instrument to examine hypothesis as an ecological analysis. The tool creates easily downloadable data and maps to conduct a multivariable analysis. Moreover, since the data is de-identified, we did not need IRB approval. Another strength is that GeoJSON specification allows for the use of multiple unrelated JavaScript libraries, such as Leaflet, Geostats, Chroma, ApexCharts, and jQuery, which permit the tool to use the best features of the available libraries.

The limitations of LionVu are that one cannot do meaningful individual analysis, uses only publicly available data, and single point-in-time information. Also, the data in Leaflet is data that must be encoded in GeoJSON format. The GeoJSON specification has no information on how to display the objects, but informal standards, such as CSS styles, can be added as properties [27,35]. Another limitation is that one has to take count data with a grain of salt and only use data that is normalized, such as by rate, percentage, or density, when using LionVu and other geospatial tools [33]. Currently, the only image format permitted in the maps are PNG image or PDF; charts are either SVG, PNG, or CSV formats, which is due to the JavaScript libraries (e.g., Leaflet Browser Print and ApexCharts), only permitting one and three file types, respectively. Finally, very often JavaScript libraries become outdated, so annual checks for any revisions to the JavaScript code are required.

### 6.4. Recommendations

Depending on community feedback, our team may include data from other Pennsylvania agencies such as Revenue, Human Services, Agriculture, Insurance, Environmental Protection, and Labor and Industry, depending on data availability. Since catchment area is a hot topic in cancer health research, our team may be able to collaborate with other agencies in different geographical areas in the future. For example, zip code data, although convenient to map, is currently not included in LionVu, because it crosses state, county, and census tract geographies, or may not have a boundary in the case of post office boxes, but may be added in the future due to its utility in specialized public health GIS applications [36], such as Florida Health Charts. We hope that future versions of Leaflet Browser Print and ApexCharts allow more file formats, such as JPEG and TIFF, which are the preferred types for most journals. Our team may explore creating bivariate maps [37], and charting for census tract data in a way that does not slow down the server. We are strongly considering the option to view multiple time periods in addition to seeing two variables side-by-side. Future releases may include created a predicted line or multivariate spatial analyses comparable to web-GIS applications, such as the Periscope Atlas developed to visualize and analyze COVID-19 data [24]. We might also explore the usage of creating a manually defined data classification that allows the end-user to change the ranges of values. A final opportunity lies in developing training courses that could count as continuing education credits for clinicians and public health researchers in geospatial thinking and reasoning skills.

## 7. Conclusions

In 2023, our team is part of the growing trend in public health research that is using specialized surveillance systems for clinical sites that include web-GIS, leading to a need for training to use the data in web-based platforms. Currently, LionVu includes 21 publicly available data sources with geographies composed of county, census tracts, health professional service areas, medically underserved areas, and select individual-level data. Our team continues to update LionVu annually to allow for mapping comparisons for a variety of different variables in each dataset for Pennsylvania. Although the geography is just Pennsylvania, it is possible based on data availability to replicate to a larger area, potentially increasing the effectiveness and efficiency of LionVu. If an organization outside of Pennsylvania needs to develop a comparable system, they can collaborate with us and can help us to develop a more powerful web-GIS tool with a server that can better manage different geographic information. Considering the massive amount of data, the hope is to create a dialog to demonstrate the potential value of web-based GIS applications to a wider audience within the public health research community.

## Supplementary Material

supplementary materials

## Figures and Tables

**Table 1.** JavaScript libraries and feedback form used in LionVu, as of 15 February 2023.

Name	Website	Description
Leaflet 1.9.3	https://leafletjs.com/	Open-source mapping JavaScript Library.
Leaflet.Browser.Print 2.0.2	https://igor-vladyka.github.io/leaflet.browser.print/	A Leaflet plugin that allows users to print full page maps directly from their browser.
Leaflet-Betterscale 2.0.1	https://daniellsu.github.io/leaflet-betterscale/	A better scalebar for Leaflet maps that is more GIS-like with alternating black/white bars.
Leaflet-control-topcenter 2.0.1	http://fcoo.github.io/leaflet-control-topcenter/demo/	Extend Leaflet to allow placement of controls in the top-center and bottom-center positions.
Leaflet.EasyButton 2.4.0	http://cliffcloud.github.io/Leaflet.EasyButton/v1/	Leaflet control buttons with icons and callbacks.
Leaflet.SvgShapeMarkers 1.4.0	http://rowanwins.github.io/Leaflet.SvgShapeMarkers/	Additional SVG marker types for Leaflet, such as triangle, diamond, and square.
Leaflet Legend 1.0.0	https://ptma.github.io/Leaflet.Legend/	Plugin for Leaflet that display legend symbols and toggles overlays.
Dom-To-Image 2.6.0	https://github.com/tsayen/dom-to-image	A JavaScript library that can turn an arbitrary DOM node into a raster PNG image.
Geostats 2.0.0	https://github.com/simogeo/geostats	A library for map classification and basic statistics.
Chroma 2.4.2	https://www.vis4.net/chromajs	A JavaScript library for color conversions and scales.
jQuery 3.6.3	https://jquery.com	A fast, small, and feature-rich JavaScript library.
jquery-fullscreen-plugin 1.1.5	https://code-lts.github.io/jquery-fullscreen-plugin	A jQuery plug-in that provides a way to control the new Fullscreen mode of modern browsers.
DataTables 1.13.1	https://www.datatables.net/download/	A jQuery plug-in that adds features to HTML tables.
Buttons 2.3.3	https://www.datatables.net/download/	A common framework for user interaction buttons.
Column Visibility 2.3.3	https://www.datatables.net/download/	End-user buttons to control column visibility.
HTML5 Export 2.3.3	https://www.datatables.net/download/	Copy to clipboard and create Excel, PDF, and CSV files from the table’s data.
ColReorder 1.6.1	https://www.datatables.net/download/	Click-and-drag column reordering.
Responsive 2.4.0	https://www.datatables.net/download/	Dynamically show and hide columns based on the browser size.
html2pdf 0.10.1	https://ekoopmans.github.io/html2pdf.js	Client-side HTML-to-PDF rendering.
ApexCharts 3.37.0	https://apexcharts.com/	A modern and interactive open-source charts library.
sweetAlerts2 11.7.1	https://sweetalert2.github.io/	An accessible replacement for JavaScript’s popular boxes.
Feedback Form	https://phppot.com/jquery/php-contact-form-with-jquery-ajax/	PHP contact form with jQuery AJAX.

**Table 2.** Data currently available in LionVu for visualization and analysis, as of 20 March 2023.

Data Type	Organization	Data Source	Topics	Year	Geography	Website
**Health Outcomes & Access to Care**	Pennsylvania Department of Health	Enterprise Data Dissemination Informatics Exchange	Age-Adjusted Cancer Mortality	2016–2020	County	https://www.phaim1.health.pa.gov/EDD/
			Age-Adjusted Cancer Incidence: Invasive, Early, & Late Stages	2015–2019	County	
		County Health Profile	Birth, Death, Cancer, Disease, Hospitalization, & Risks	2022	County	https://www.health.pa.gov/topics/HealthStatistics
		Cancer Survival	Cancer 5 Year Net Survival	2021	County	
		Healthcare Facilities and Licensing	Ambulatory Surgery Centers	2022	Point	http://www.pasda.psu.edu
			Hospitals	2022	Point	
			Rural Health Centers	2022	Point	
	Food & Drug Administration	Mammography Facility Database	Mammography Locations	2020	Point	https://www.fda.gov
	Penn State Health	Locations	Inpatient, Outpatient, & Outreach Sites	2022	Point	https://www.pennstatehealth.org/locations
	Health Resources & Services Administration	Health Underserved Areas	Dental, Medical, Mental Health, & Physical Health	2022	Various	https://data.hrsa.gov/data/download
		Health Center Service Delivery & Look-Alike Sites	Federally Qualified Health Centers & Look-Alike Locations	2022	Point	
**Socioeconomic & Environmental Exposures**	United States Census Bureau	American Community Survey	Demographics, Social, Economic, & Housing	2017–2021	County	https://data.census.gov
		Small Area Health Insurance Estimates	Health Insurance by Age, Race, Sex, & Income	2020	County	https://www.census.gov/programs-surveys/sahie.html
	Environmental Protection Agency	Environmental Justice Screening Mapping Tool	Environmental & Socioeconomic Indicators	2022	Census Tract	https://www.epa.gov/ejscreen/
	Agency for Toxic Substances & Disease Registry	Social Vulnerability Index	Socioeconomics, Household, Racial Ethnic Minorities, Housing, & Transportation	2020	Census Tract	https://www.atsdr.cdc.gov/placeandhealth/svi/
**Multiple**	County Health Rankings & Roadmaps	County Health Ranking	Health Outcomes, Behaviors, Clinical Care, Socioeconomic, & Environmental	2022	County	https://www.countyhealthrankings.org
**Geographical**	United States Census Bureau	TIGER/Line^®^ Shapefile	County or Census Tract Geographies; Land & Water Areas	2022	County Census Tract	https://www.census.gov/geographies.html
	Appalachian Regional Commission	Appalachian Subregions	County Boundaries Filtered by Appalachian Subregion & Catchment Area, Pennsylvania	2021	Line String	https://www.arc.gov

**Table 3.** Comparison of ten web-GIS public health tools, as of 26 March 2023.

Name	Website	Geography	Health Condition
LionVu	https://app-phs.hmc.psu.edu/lionvu/	Pennsylvania	Health related data
Enterprise Data Dissemination Informatics Exchange	https://www.phaim1.health.pa.gov/EDD/	Pennsylvania	Health related data
CDC: United States Cancer Statistics	https://gis.cdc.gov/Cancer/USCS	USA	Cancer
CDC: Diabetes Public Health Resource	https://www.cdc.gov/diabetes	USA	Diabetes
CDC: Division for Heart Disease and Stroke Prevention	https://www.cdc.gov/dhdsp	USA	Heart disease stroke
CDC: Division of Oral Health	https://www.cdc.gov/OralHealth	USA	Oral health
EpiScanGIS	https://www.episcangis.org/	Germany	Meningococcal
HealthMap	https://www.healthmap.org	Global	COVID-19, influenza
Kentucky Cancer Registry	https://www.cancer-rates.info/ky/	Kentucky	Cancer
Florida Health Charts	https://www.flhealthcharts.gov/	Florida	Health related data
Periscope Atlas	https://www.periscopeproject.eu	Europe	COVID-19

## Data Availability

All available datasets integrated inside LionVu and cited in this paper (i.e., Table 2), and found in Supplementary Material S2, accessed on 27 February 2023.

## Abbreviations

ArcGIS: software developed and maintained by Esri (Environmental Systems Research Institute) since 1999
CDC: Centers for Disease Control and Prevention
CSS: Cascading Style Sheet
CSV: Comma Separated Values file format, also the name of a Python library
EJSCREEN: Environmental Justice Screening and Mapping Tool, EPA data set that provides census tract information on national environmental justice issues
EPA: Environment Protection Agency
FDA: Food and Drug Administration
GeoJSON: Geographical JavaScript Object Notation, a OGS (Open Geospatial Consortium) format for encoding geographical features and attributes based on JSON
GeoDa: Introductory spatial data analysis software, which does basic spatial analysis like spatial autocorrelation
GEOID: Geographic Identifiers, are numeric codes that uniquely identify all administrative/legal and statistical geographic areas for which the Census Bureau tabulates data
GIS: Geographical Information Systems
HIPAA: Health Insurance Portability and Accountability Act of 1996, protects health insurance data with PHI
HRSA: Health Resources and Services Administration
HTML: Hypertext Markup Language
IRB: Institutional Review Board, reviews the ethics of human-subject research
JPEG: Joint photographic expert’s group, a file format that involves lossy compression, where information is lost after compression
JSON: JavaScript Object Notation, a lightweight, text-based, language-independent data interchange format, based on the JavaScript programming language
KML: Keyhole Markup Language
NAD83: North American Datum of 1983
NCI: National Cancer Institute
NIH: National Institutes of Health, which include NCI
OS: Operating System Python Library
P30: NIH grant for Core Centers or Centers of Excellence
PDF: Portable Document Format
PHI: Personally Identifiable Health Information, including most geographies less than state
PHP: Hypertext Preprocessor, a server-based language that permits the sending of emails from the feedback form
PNG: Portable Network Graphic image file, a lossless compression that does not lose information
PyQt5: Python library with dual licenses on all platforms under the Riverbank Commercial License and the GPL (general public license) v3.
QGIS: Quantum GIS software
R: free software environment for statistical computing and graphics
RUCC: Rural-Urban Continuum Code
RE: Regular Expression Python Library, USDA (United States Department of Agriculture) classification for rurality, 2013
SAS 9.4: Statistical Analysis System software version 9.4
SHP: shapefile
SVG: Scalable Vector Graphics, XML-based format with 2-D vector graphics
TIFF: tagged image file format, can be lossy or lossless with high file size
TIGER: Topologically Integrated Geographic Encoding and Referencing, United State Census Bureau, 2022
WGS84: World Geodetic System Datum of 1984
