# Supplementary material for: LionVu: A Data-Driven Geographical Web-GIS Tool for Community Health and Decision-Making in a Catchment Area
Source: Geographies. Author manuscript; Available in PMC 2023 Nov 22. (PMC10665118; doi:10.3390/geographies3020015)
Supplement: supplementary materials [file NIHMS1943956-supplement-supplementary_materials.zip › S1_data_leafletjs.zip!/data_leafletjs/README.html]

### Plugin Builder Results

Congratulations! You just built a plugin for QGIS!  
  

Your plugin **dataLeafletJS** was created in:  
  **C:/Users/Owner.Owner-PC/AppData/Roaming/QGIS/QGIS3/profiles/default/python/plugins\data\_leafletjs**

Your QGIS plugin directory is located at:  
  **C:/Users/Owner.Owner-PC/AppData/Roaming/QGIS/QGIS3/profiles/default/python/plugins**

### What's Next

1. In your plugin directory, compile the resources file using pyrcc5 (simply run **make** if you have automake or use **pb\_tool**)- Test the generated sources using **make test** (or run tests from your IDE)- Copy the entire directory containing your new plugin to the QGIS plugin directory (see Notes below)- Test the plugin by enabling it in the QGIS plugin manager- Customize it by editing the implementation file **data\_leafletjs.py**- Create your own custom icon, replacing the default **icon.png**- Modify your user interface by opening **data\_leafletjs\_dockwidget\_base.ui** in Qt Designer

Notes:

- You can use the **Makefile** to compile and deploy when you
  make changes. This requires GNU make (gmake). The Makefile is ready to use, however you
  will have to edit it to add addional Python source files, dialogs, and translations.- You can also use **pb\_tool** to compile and deploy your plugin. Tweak the *pb\_tool.cfg* file included with your plugin as you add files. Install **pb\_tool** using
    *pip* or *easy\_install*. See **http://loc8.cc/pb\_tool** for more information.

For information on writing PyQGIS code, see **http://loc8.cc/pyqgis\_resources** for a list of resources.

©2011-2018 GeoApt LLC - geoapt.com
